# Neuroendocrine Carcinomas of the Canine Mammary Gland: Histopathological and Immunohistochemical Characteristics

**DOI:** 10.3389/fvets.2020.621714

**Published:** 2021-01-05

**Authors:** Karen Yumi Ribeiro Nakagaki, Maíra Meira Nunes, Ana Paula Vargas Garcia, Marina De Brot, Geovanni Dantas Cassali

**Affiliations:** ^1^Laboratory of Comparative Pathology, Department of General Pathology, Institute of Biological Sciences, Federal University of Minas Gerais, Belo Horizonte, Brazil; ^2^Department of Anatomic Pathology, A. C. Camargo Cancer Center, São Paulo, Brazil

**Keywords:** diagnosis, female dog, histological classification, solid carcinoma, neuroendocrine carcinoma, mammary cancer

## Abstract

Invasive mammary carcinomas with neuroendocrine differentiation are rare in women and were reported only once in female dogs. For the present study, ten cases of solid mammary carcinoma positive for chromogramin A in immunohistochemistry were selected. Histopathological characteristics of these tumors were described and immunohistochemical evaluation was performed with chromogranin A, synaptophysin, CD56, NSE, PGP 9.5, pancitokeratin, Ki67, estrogen receptor (ER), and progesterone receptor (PR). The average animal age was 13.2 years old and the average tumor size was 4.8 cm. In total, 70% of the neoplasms were classified as grade III and 30% as grade II by the Nottingham histological grade system. High mitotic index was observed with a mean of 27.5 mitoses in 10 high magnification fields. Only one case showed typical carcinoid tumor characteristics. In addition, vascular invasion was shown in 3 tumors. All carcinomas were positive for chromogran A, while only two cases were reactive to synaptophysin. For PGP 9.2, NSE and CD56, we observed positivity of 100, 90, and 70%, respectively, in the samples, being that no tumor was positive for all the neuroendocrine markers. All neoplasms showed ER and PR in at least 10% of neoplastic cells, while Ki67 varied from 29 to 95%, with mean mitotic index of 67%. Four of the ten animals died within 1 year of the tumor diagnosis. Neuroendocrine neoplasms occur in the canine mammary gland and are propably underdiagnosed. This is due to their non-specific morphological characteristics and the low use of neuroendocrine immunohistochemistric markers the diagnostic routine. More studies are necessary to determine the prognosis of this new histological type.

## Introduction

Neuroendocrine tumors are a group of biologically and clinically heterogeneous neoplasms that originate most commonly in lungs, gastrointestinal tract and pancreas (1, 2). Although their occurrence is rare, pure neuroendocrine tumors and invasive breast carcinomas with neuroendocrine features have already been reported in women (3–5), while only one case has been reported in a female dog (6).

These tumors were first recognized in women in 1963 by Feyrter and Hartmann (7) based on a “carcinoid” growth pattern seen in two cases of invasive breast carcinoma. Later, in 1977, Cubilla and Woodruff (8) described eight cases of breast cancer showing a growth pattern typical of a “carcinoid tumor,” and several recent reviews have since been published (9–12).

Finally, the classification of breast tumors of the World Health Organization (WHO) in 2003 recognized neuroendocrine carcinomas of the breast as a special histological type of invasive carcinoma in which more than 50% of the neoplastic cells express at least one neuroendocrine marker (13). Next, the 2012 classification included a chapter on “Carcinomas with neuroendocrine features,” in which the minimum cut-off of tumor cells with positive labeling for neuroendocrine markers was removed (9, 14).

The most recent WHO classification (2019) (15) categorizes breast cancers with neuroendocrine differentiation in three groups: (1) invasive carcinoma with neuroendocrine differentiation; (2) neuroendocrine tumor (NET); (3) neuroendocrine carcinoma (NEC). However, the neuroendocrine differentiation detected by either histochemical or immunohistochemical analysis may be seen in 10–30% of invasive breast carcinomas of no special type (IBC NST). Additionally, special types of breast cancer may also show expression of neuroendocrine markers, particularly solid papillary carcinomas and mucinous carcinomas, and should not be classified as NETs or NECs. When neuroendocrine morphologic characteristics and neuroendocrine marker expression are focal or are not distinct enough to classify a neoplasm as NET or NEC, an IBC NST with neuroendocrine differentiation must be considered. Notably, most breast cancers with neuroendocrine differentiation belong in the first group, as pure neuroendocrine tumors of the breast are exceptionally rare.

Neuroendocrine tumors of the breast correspond to an invasive neoplasm composed of densely cellular solid nests or trabeculae of cells, usually with a low to intermediate grade morphology, separated by delicate fibrovascular stroma. Papillary, insular and alveolar-like patterns may be seen. On the other hand, neuroendocrine carcinomas are invasive carcinomas characterized by the proliferation of small or large, high-grade neoplastic cells (small cell neuroendocrine carcinoma and large cell neuroendocrine carcinoma, respectively). Both subtypes present neuroendocrine morphological features, cytoplasmic neurosecretory granules and uniform immunohistochemical positivity for neuroendocrine markers (15).

Data on breast cancers with neuroendocrine differentiation are limited. Also, the true incidence of neuroendocrine neoplasms of the breast is difficult to evaluate, because many of the classic histopathological features of neuroendocrine tumors that occur in other organs are not present in their breast counterpart (13, 16). In addition, neuroendocrine markers are not routinely tested in invasive breast carcinomas, since there is no clinical relevance of neuroendocrine differentiation as an individual characteristic (12, 15).

In the female dog, the classification of mammary neoplasms is mainly based on the histopathological pattern and, to a lesser extent, on the histogenetic classification, due to the difficulty in determining the origin of a specific cell type in certain mammary tumors (17). Among the histological types described, the solid carcinoma is a common pattern of canine mammary tumor that presents some variations in cell characteristics, which makes many researchers believe that there may be several origins for these cells (18–20).

In this study, we aimed to investigate the presence of neuroendocrine differentiation in 10 cases of solid mammary carcinoma of female dogs, in order to promote a greater recognition and appropriate classification of this histological type in this species.

## Materials and Methods

### Ethics Statement

The study was approved by the ethics committee on animal use (CEUA/UFMG) under protocol number 11/2017, on June 5th, 2017.

### Animals

Ten cases of canine mammary solid carcinoma, positive for chromogramin A in immunohistochemistry, were selected for this study. The samples were from the Laboratory of Comparative Pathology of Minas Gerais Federal University (UFMG).

### Histopathology

Representative samples of tumors removed by incisional or excisional biopsy were obtained and included in paraffin blocks. Consecutive histologic sections were prepared and stained by the hematoxylin and eosin routine method. Neoplasm slides were evaluated and diagnoses were defined according to the “Consensus for the diagnosis, prognosis, and treatment of canine mammary tumors-2013” (20). The Nottingham histologic grade system was applied to determine tumor grade (21).

### Immunohistochemistry

Sections of 4 μm thickness from primary tumors were prepared and mounted on common slides for IHC analysis. The antigen was immunodetected by the detection system anti-mouse/anti-rabbit (Novolink Polymer Detection System, Leica Biosystems, Newcastle Upon Tyne, United Kingdom) according to the manufacturer's instructions. The endogenous peroxidase activity was blocked with a 10% hydrogen peroxide (H_2_O_2_) solution in methyl alcohol. Reagents were manually applied and immunoreactivity was visualized by incubating the slides with diaminobenzidine chromogen (DAB Substrate System, Dako, Carpinteria, CA, USA) for 3 min. Details of the antibodies against Synaptophysin (1, 22, 23), NSE (22, 24), CD56 (24), PGP 9.5 (25, 26), Chromogranin A (25, 27), Estrogen Receptor - RE (28), Progesterone Receptor - PR (29), Pancitokeratin (22) and Ki67 (30), dilutions, antigen retrieval procedures and incubation times used in the immunostaining process are shown in Table 1. Normal canine mammary gland was used as an internal positive control for Estrogen and Progesterone Receptors and Pancitokeratin. For Synaptophysin, NSE, CD56, PGP 9.5 and Chromogranin A, canine adrenal gland was used as positive control. Negative controls were performed using a normal serum (Lab Vision Ultra V Block) in place of the primary antibody.

**Table 1:** Antibodies, dilutions, incubation time and temperature and methods of antigenic recovery for the immunohistochemical reactions.

**Antibody**	**Manufacturer**	**Clone**	**Dilution**	**Incubation time/temperature**	**Antigenic recovery method**
Synaptophysin	Monosan	SY38	1:100	Overnight 4°C	10 mM citrate (pH 6.0) in the pressure cooker (Pascal^R^, Dako)
NSE	Dako	BBS/NC/VI-H14	1:1000	Overnight 4°C	10 mM citrate (pH 6.0) in the pressure cooker (Pascal^R^, Dako)
CD56	Biocare Medical	BC56C04	1:50	Overnight 4°C	10 mM citrate (pH 6.0) in the pressure cooker (Pascal^R^, Dako)
PGP 9.5	Cell Marque	polyclonal	1:500	Overnight 4°C	10 mM citrate (pH 6.0) in the pressure cooker (Pascal^R^, Dako)
Chromogranin A	Dako	DAK-A3	1:100	Overnight 4°C	10 mM citrate (pH 6.0) in the pressure cooker (Pascal^R^, Dako)
Estrogen Receptor (RE)	Dako	1D5	1:50	Overnight 4°C	10 mM citrate (pH 6.0) in the pressure cooker (Pascal^R^, Dako)
Progesterone receptor (RP)	NeoMarkers	Ab-1 (hPRa2)	1:50	Overnight 4°C	10 mM citrate (pH 6.0) in the pressure cooker (Pascal^R^, Dako)
Pancitokeratin	Dako	AE1/AE3	1:500	Overnight 4°C	10 mM citrate (pH 6.0) in the pressure cooker (Pascal^R^, Dako)
Ki67	Dako	MIB-1	1:50	Overnight 4°C	10 mM citrate (pH 6.0) in the pressure cooker (Pascal^R^, Dako)

### Immunohistochemical Evaluation

The cell proliferation index (Ki67) was calculated by manually counting the number of positive nuclei in a total of 500 neoplastic cells in areas with the highest levels of positivity (hot spot/hot zones) through image J software (National Institute of Health, Bethesda, Maryland, USA). A 20% score was used as cutoff point to classify cases with high or low proliferation index. Positivity for estrogen receptor (ER) and progesterone receptor (PR) was defined as the presence of nuclear expression in >10% of neoplastic cells (3). The Expression of the neuroendocrine markers chromogranin A, synaptophysin, CD56, NSE and PGP 1.9 was assessed. The evaluation pancytokeratin expression (AE1/AE3) was qualitative and classified as positive positive when there was cytoplasmic staining of neoplastic cells.

## Results

The mean age of the ten studied animals was 13.2 years old, ranging from 9 to 16 years old. Mean tumor size was 4.8 cm, excluding one case of incisional biopsy for which the actual size of the neoplasm was not informed. The main pathological parameters are detaleid in Table 2.

**Table 2:** Macroscopic and histopathological characteristics of mammary neoplasms with neuroendocrine differentiation.

**Patients**	**Tumor size measured to its largest extent (cm)**	**Mitotic figures in 10 HPFs**	**Histological grade**	**Presence of lymphatic invasion**	**Regional lymph node**
Patient 1	8.0	43	III	No	Not analyzed
Patient 2	1.5^*^	46	III	Yes	Not analyzed
Patient 3	8.0	25	III	No	No metastasis
Patient 4	2.0	18	II	Yes	With metastasis
Patient 5	3.0	41	III	No	Not analyzed
Patient 6	5.0	34	III	No	Not analyzed
Patient 7	4.5	22	III	No	Not analyzed
Patient 8	7.0	22	III	No	No metastasis
Patient 9	2.0	11	II	No	No metastasis
Patient 10	4.3	13	II	Yes	Not analyzed

* *HPFs, high-power fields*.

In general, tumors showed a high mitotic count, with a mean number of 27.5 mitoses in 10 high-power fields (40X). Seventy percent (7/10) of the neoplasms were classified as grade III and 30% (3/10) as grade II. Lymph nodes of only 4 cases were referred for analysis, of which one presented metastasis on histopathological examination.

**Figure 1:**
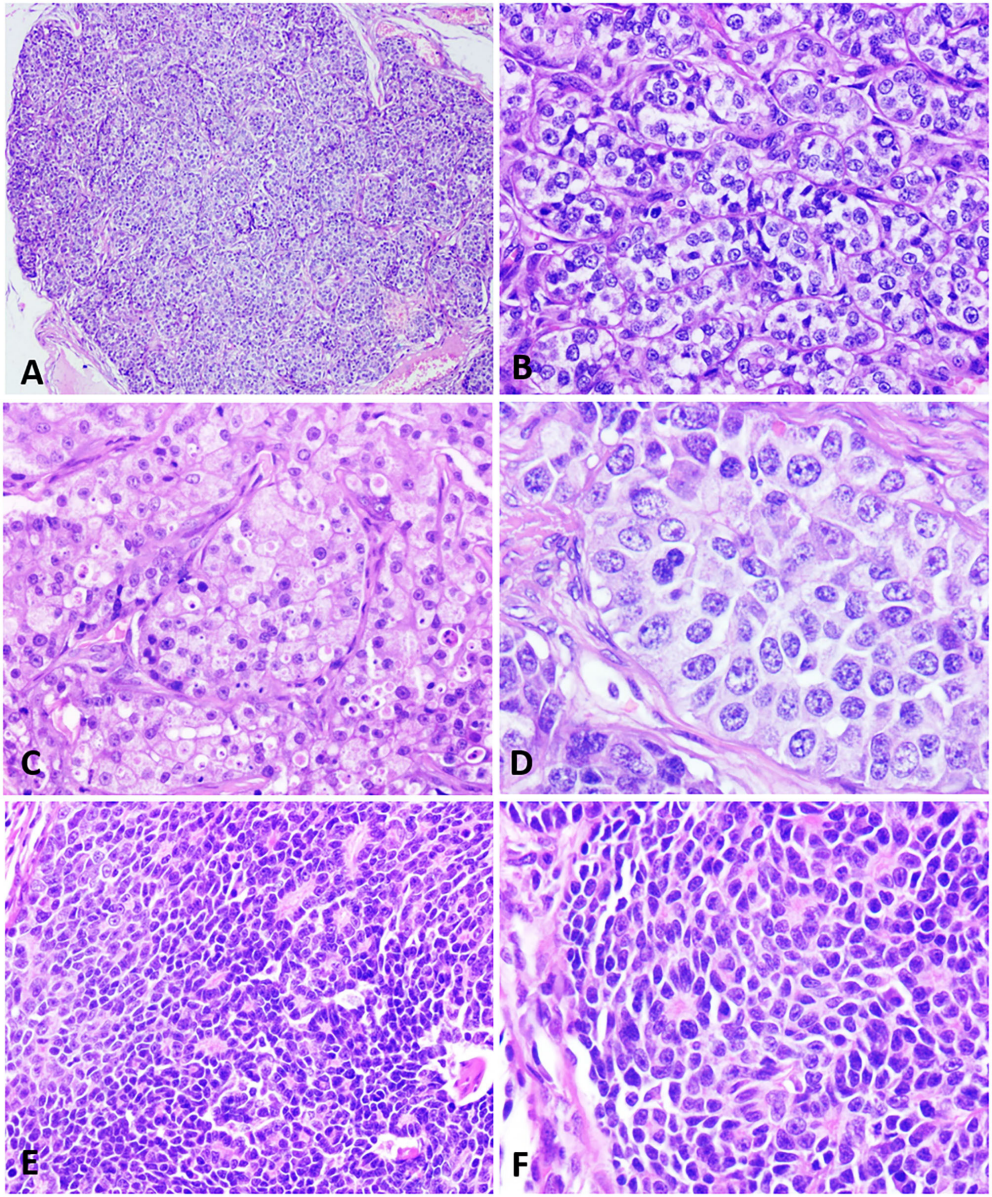
Histopathological characteristics of neuroendocrine carcinomas in the female dog. **(A)** A mammary lump showing a solid arrangement. Hematoxylin and eosin.10x. **(B)** Solid nests of neoplastic cells separated by a delicate fibrovascular stroma. Hematoxylin and eosin.40x. **(C)** Cells with a finely granular eosinophilic cytoplasm, sometimes displaying intracytoplasmic vacuoles. Hematoxylin and eosin.40x. **(D)** Neoplastic cells with round to oval nuclei, finely dotted chromatin, and conspicuous nucleoli. Hematoxylin and eosin.60x. **(E)** Neoplastic cells disposed in a solid arrangement, occasionally in palisades and forming rosettes. Hematoxylin and eosin.40x. **(F)** Cells exhibiting a scarce, slightly eosinophilic cytoplasm, with small and hyperchromatic nuclei. Hematoxylin and eosin.40x.

Similar morphological patterns were observed in the histopathological analysis of all cases, with 100% demonstrating a solid cell arrangement (Figure 1A) and at least some proliferation areas *in situ*. Most tumors exhibited an infiltrative growth pattern with invasion into the dermis and adjacent adipose tissue, although a circumscribed pattern was seen in some cases. Sixty percent of the cases (6/10) exhibited arrangement in small solid nests of cells delimited by delicate fibrovascular stroma (Figure 1B). Neoplastic cells presented a cytoplasm of moderate size, slightly eosnophilic, with varying degrees of fine granulation, or a clear cytoplasm, with vacuolization (Figure 1C). The nuclei were large, round to oval, with varying degrees of atypia. In addition, 60, 30, and 10% of the cases showed marked, moderate and mild anisocariosis, respectively. Nuclear chromatin was slightly dotted and dispersed (salt and pepper aspect) in most cases, and sometimes with fine condensation along the nuclear membrane. The nucleoli were either small and multiple or sometimes single and large (Figure 1D).

One of the neoplasms (patient 4) showed a distinct, peculiar morphology, with cells arranged in large coalescing solid nests, containing palisade cells, often forming rosettes (Figure 1E). The cytoplasm was scarce, slightly eosinophilic, with oval nuclei, small, hyperchromatic and unique nucleoli or occasionally prominent (Figure 1F). This morphological pattern was compatible with the histologic characteristics of carcinoid tumors.

Lymphovascular invasion was identified in 3 of the 10 cases (30%). Areas of necrosis, mainly at the center of solid nests, were observed in 70% of the neoplasms.

**Figure 2:**
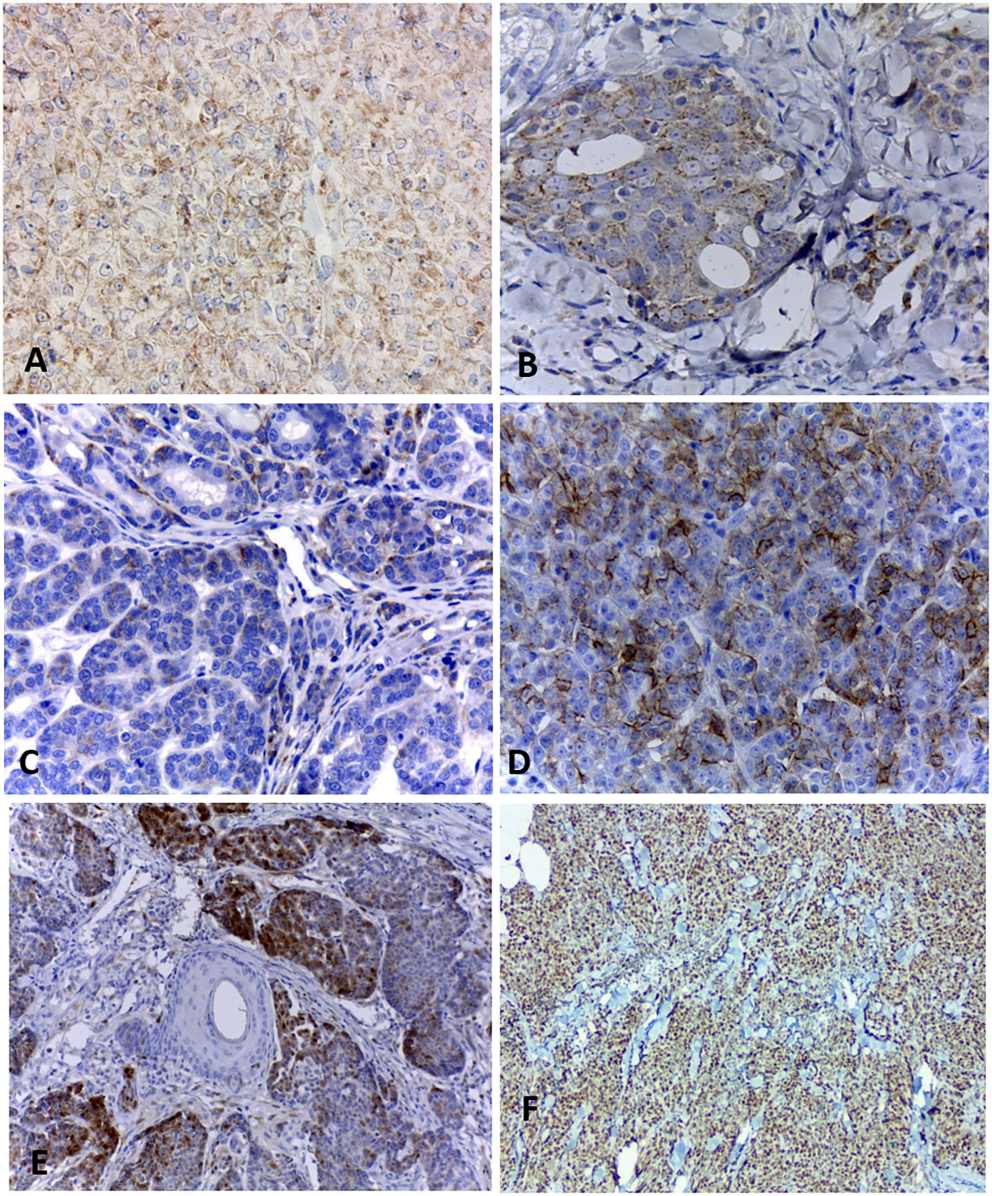
Immunohistochemical staining of canine mammary solid carcinomas. **(A)** Neoplastic cells showing positive cytoplasmic staining for chromogranin A, with a granular pattern. 40x. **(B)** Cytoplasmic expression of synaptophysin in more than 50% of neoplastic cells. 40x. **(C)** Less than 50% of positive neoplastic cells for CD56. 40x. **(D)** Expression of NSE in more than 50% of neoplastic cells. 40x. **(E)** Multifocal staining for PGP 9.5 in 10% of neoplastic epithelial cells. **(F)** Nuclear positivity Ki67 in 95% of neoplastic cells. 10x.

Immunohistochemistry results are shown in Table 3. All cases were positive for chromogranin A (Figure 2A) and the cytoplasmic staining was granular with variable intensity. Only two cases were positive for synaptophysin (Figure 2B). Seven tumors (7/10) were positive for CD56 (Figure 2C), while nine (9/10) were positive for NSE (Figure 2D). All cases were positive for PGP 9.5 (Figure 2E). No case was positive for all neuroendocrines.

**Table 3:** Expression of chromogranin A, synaptophysin, NSE, CD56, PGP 9.5, Ki67, ER, PR and pancitokeratin in solid mammary carcinomas with neuroendocrine features.

**Patients**	**Chromogranin A**	**Synaptophysin**	**NSE**	**CD56**	**PGP 9.5**	**Ki67**	**ER**	**PR**	**CK AE1/AE3**
Patient 1	Positive	Negative	Positive	Negative	Positive	50%	10–25%	>75%	Positive
Patient 2	Positive	Positive	Positive	Negative	Positive	95%	51–75%	>75%	Positive
Patient 3	Positive	Negative	Positive	Positive	Positive	75%	51–75%	>75%	Positive
Patient 4	Positive	Positive	Negative	Positive	Positive	83%	51–75%	>75%	Positive
Patient 5	Positive	Negative	Positive	Positive	Positive	50%	10–25%	>75%	Positive
Patient 6	Positive	Negative	Positive	Positive	Positive	90%	25–50%	>75%	Positive
Patient 7	Positive	Negative	Positive	Positive	Positive	89%	25–50%	>75%	Positive
Patient 8	Positive	Negative	Positive	Positive	Positive	29%	10–25%	>75	Positive
Patient 9	Positive	Negative	Positive	Positive	Positive	56%	10–25%	51–75%	Positive
Patient 10	Positive	Negative	Positive	Negative	Positive	54%	10–25%	51–75%	Positive

All neoplasms expressed estrogen and progesterone receptors in at least 10% of neoplastic cells and were positive for pancytokeratin (AE1/AE3). Ki67 (Figure 2F) was considered high in most carcinomas, with a mean of 67% of positive cells, ranging from 29 to 95%.

Four of the 10 studied animals (patients 1, 2, 6, and 10) died or were euthanized within 1 year of diagnosis due to the neoplasm development. Two animals (patients 3 and 4) died due to other causes unrelated to the tumor and two patients (8 and 9) are still alive with no sings of recurrence and metastasis after 1 year and 6 months and 3 years and 8 months of diagnosis, respectively. The follow-up of patients 5 and 7 after surgery was not feasible. Only one of the eight patients with follow-up (patient 10) received complementary treatment after surgery.

## Discussion

Invasive carcinomas with neuroendocrine differentiation of the human breast are under-recognized in the practical routine and represent 0.5–1% of all breast cancers (11, 12, 15). In veterinary medicine, this histological type is not yet well recognized, with a single prior case report in the literature (6), making ours the first retrospective study on neuroendocrine carcinomas in the female dog.

The histogenesis of neuroendocrine tumors of the breast is debatable mainly due to the difficulty in locating neuroendocrine cells in normal mammary glands (16, 31). Viacava et al. (31) did not find histochemical, immunohistochemical and ultrastructural evidence of neuroendocrine differentiation in normal cells of the fetal and adult mammary glands in their study, indicating that this differentiation may happen in the process of tumor progression.

From a clinical point of view, the importance of neuroendocrine differentiation in invasive breast carcinomas is not well stablished. While some studies have stated that there is no prognostic value, others have shown that it is associated to a better or worse prognosis (14, 16, 32). Sapino et al. (33) concluded that the histological grade greatly influenced the clinical evolution of neuroendocrine carcinomas of the breast. Poorly differentiated, grade III neuroendocrine carcinomas with a high proliferative activity behaved aggressively. On the other hand, patients with well-differentiated, grade I tumors with a low proliferative index remained alive after more than 13 years of follow-up. Therefore, the impact of neuroendocrine differentiation on prognosis remains unclear and may be explained by the heterogeneous nature of tumors that fall into this category, as this group includes special breast cancer types with a low-grade morphology and indolent clinical course, as well as high-grade aggressive carcinomas (32, 34). Of the eight followed-up patients, four died due to the unfavorable neoplasm clinical evolution in <1 year of diagnosis. In this sense, the fact that half of these followed-up patients survived for less than an year after diagnosis may lead to the conclusion that this type of carcinoma presents a guarded to poor prognosis compared to other carcinomas with better prognosis in female dogs, such as carcinoma in mixed tumor, which does not reach the survival median until 2 years of follow-up (35).

The diagnosis of carcinoma with neuroendocrine differentiation based solely on morphologic characterisitics is a challenge and not feasible most of the times, once many of the classic histologic features of neuroendocrine carcinomas that occur in other organs are not present in neuroendocrine carcinomas of the mammary gland (13, 16, 36). Thus, the morphologic features found in this study were similar to those described in other studies that confirmed the presence of neuroendocrine differentiation by immunohistochemistry (11, 14, 32, 37). Only one case exhibited a typical morphology of a carcinoid tumor, with smaller cells, sometimes forming rosettes, hyperchromatic nuclei and scarce cytoplasm, analogous to carcinoid tumors and well-differentiated neuroendocrine carcinomas previously reported in the human breast and other organs (38, 39). According to the WHO classification of breast tumors of 2019, this sole case should be categorized as a neuroendocrine tumor, whereas the other nine tumors should be classified as large cells neuroendocrine carcinomas according to morphologic and immunohistochemical characteristics (15).

Metastases from other neoplasms in women's breast are uncommon and metastases of neuroendocrine carcinoma are even rarer, representing 1–2% of all metastatic tumors of the breast (37). However, the possibility of metastatic neuroendocrine carcinomas should always be excluded with adequate clinical and radiological examinations as well as immunohistochemical studies to attest a breast origin in order to diagnose a primary invasive breast carcinoma with neuroendocrine features (14). The presence of an associated carcinoma *in situ* and positivity for hormonal receptors may also be useful for the diferential diagnosis (14, 16, 37, 40, 41). The animals studied herein had no history of neoplasms in other locations, but imaging data were not obtained. All tumors were positive for ER and PR, and had proliferation areas *in situ*, corroborating the hypothesis that these were primary mammary lesions.

Among neuroendocrine differentiation markers, chromogranin A and synaptophysin are the most frequently used (1, 34). In our study, only 2 of 10 cases showed positivity for both markers and similar results have been reported by Wachter et al. (13). Such findings highlight the importance of a panel including at least two markers in cases suspected for neuroendocrine carcinoma, since these tumors will not always be positive for both antibodies.

Our findings show that neuroendocrine carcinomas occur in the canine mammary gland, as well as in the human breast and may be underdiagnosed when they are included in the group of solid carcinomas. However, a definitive diagnosis based on histopathological examination alone is challenging, stressing the need of using specific markers for neuroendocrine differentiation such as chromogranin and synaptophysin for confirmation. Thus, complementary studies with clinical and therapeutic follow-up are essential to define the prognosis of this new histological type, in addition to establishing implications in target therapy responsiveness.

## Data Availability Statement

The raw data supporting the conclusions of this article will be made available by the authors, without undue reservation.

## Ethics Statement

The animal study was reviewed and approved by CEUA - UFMG. Written informed consent was obtained from the owners for the participation of their animals in this study.

## Author Contributions

KN and MN collected patient data and samples from the UFMG Laboratory of Comparative Pathology file. KN, AG, and GC performed histopathological and immunohistochemical analyses. KN, GC, and MD participated in the writing of the manuscript. All authors contributed to the article and approved the submitted version.

## Conflict of Interest

The authors declare that the research was conducted in the absence of any commercial or financial relationships that could be construed as a potential conflict of interest.
